# Analysis of leukocyte membrane protein interactions using protein microarrays

**DOI:** 10.1186/1471-2091-6-2

**Published:** 2005-03-01

**Authors:** Michelle Letarte, Despina Voulgaraki, Deborah Hatherley, Mildred Foster-Cuevas, Nigel J Saunders, A Neil Barclay

**Affiliations:** 1 Sir William Dunn School of Pathology, University of Oxford, Oxford, OX1 3RE, UK; 2 Cancer Research Program, Hospital for Sick Children, Toronto, Ontario, M5G 1X8, Canada

## Abstract

**Background:**

Protein microarrays represent an emerging class of proteomic tools to investigate multiple protein-protein interactions in parallel. A sufficient proportion of immobilized proteins must maintain an active conformation and an orientation that allows for the sensitive and specific detection of antibody and ligand binding. In order to establish protein array technology for the characterization of the weak interactions between leukocyte membrane proteins, we selected the human leukocyte membrane protein CD200 (OX2) and its cell surface receptor (hCD200R) as a model system. As antibody-antigen reactions are generally of higher affinity than receptor-ligand binding, we first analyzed the reactivity of monoclonal antibodies (mAb) to normal and mutant forms of immobilized CD200R.

**Results:**

Fluorescently labelled mAb DX147, DX136 and OX108 were specifically reactive with immobilized recombinant hCD200R extracellular region, over a range of 0.1–40 μg ml^-1 ^corresponding to a limit of sensitivity of 0.01–0.05 femtomol per spot. Orientating hCD200R using capture antibodies, showed that DX147 reacts with an epitope spatially distinct from the more closely related DX136 and OX108 epitopes. A panel of soluble recombinant proteins with mutations in hCD200R domain 1 produced by transiently transfected cells, was arrayed directly without purification and screened for binding to the three mAb. Several showed decreased binding to the blocking mAb DX136 and OX108, suggesting close proximity of these epitopes to the CD200 binding site. Binding of hCD200 to directly immobilized rat, mouse, and hCD200R was achieved with multimeric ligands, in the form of biotinylated-hCD200 coupled to FITC-labelled avidin coated beads.

**Conclusion:**

We have achieved sensitive, specific and reproducible detection of immobilized CD200R with different antibodies and mapped antigenic epitopes for two mAb in the vicinity of the ligand binding site using protein microarrays. We also detected CD200 binding to its receptor, a low affinity interaction, using beads presenting multivalent ligands. Our results demonstrate the quantitative aspects of protein arrays and their potential use in detecting simultaneously multiple protein-protein interactions and in particular the weak interactions found between leukocyte membrane proteins.

## Background

Protein-protein interactions are fundamental to biological processes and their analysis is essential for the understanding of cellular pathways. Given the complexity and the dynamic range of the proteome, estimated at 10^7 ^proteins, the elucidation of protein interactions requires the development of comprehensive, high-throughput proteomic methods that allow quantification of multiple proteins simultaneously [1,2]. The development of protein microarrays represents an attractive new high-throughput technology platform. It involves the printing of ordered arrays of biomolecules onto a solid surface in miniaturized format that allows for the simultaneous determination of multiple interactions using small amounts of samples within a single experiment. The basic principles for highly sensitive "microspot" ligand-binding assays were described by Ekins [3,4] who proposed the "ambient analyte theory" and showed that microspots containing small amounts of capture molecules were able to detect low analyte concentrations with very high accuracy and sensitivity. Since then, miniaturized protein arrays are emerging as one of the most powerful proteomics tools but their application is far more complex [5] than the DNA microarrays (reviewed in [6-8]) due to structural complexity and heterogeneity of proteins, including their post-translational modifications. Binding of the proteins onto the solid surface of an array must maintain tertiary structure sufficient for functions such as receptor-ligand binding or antibody reactivity. Chemically derivatized microarray surfaces [9,10] or the use of mAb [11,12] have been shown to maintain protein functionality, thus increasing the potential for successful application of microarray technology in proteomics.

The study of leukocyte membrane protein interactions provides a particular need because of the large number of interactions yet to be defined [13,14] and a technical challenge as these interactions are often of very low affinity with K_D _in the range 1–200 μM [15,16]. Although weak, these interactions are important in the context of leukocytes interacting with other cells as illustrated by all the functional data on the interaction of CD8 with MHC Class II (K_D _= 200 μM) [17]. The proteins involved usually contain folded domains, the most common type belonging to the immunoglobulin superfamily (IgSF) [13]. Such domains often interact through large faces of the proteins and require proper folding [18,19]. When measuring low affinity interactions, misleading results can be obtained from unfolded or aggregated materials which are not really a problem when dealing with high affinity interactions such as with cytokines and their receptors, or between proteins and linear epitopes such as lectins and carbohydrates. In addition many leukocyte surface proteins are heavily glycosylated and the oligosaccharides, even if not directly involved in binding, may be important in maintaining biologically active proteins [20]. Thus, in applying the protein microarray technology to the study of leukocyte surface protein interactions, it is imperative that the proteins are expressed in eukaryotic systems to ensure correct disulphide bond formation and post-translational modifications.

In this study we chose a well characterized interaction between CD200 (previously called OX2) and its receptor CD200R (reviewed in [21]) as a model system to devise a high throughput protein array method for characterization of the interactions between leukocyte surface proteins. CD200 is a widely distributed membrane protein with two extracellular IgSF domains and a short cytoplasmic region unlikely to signal. It interacts with a receptor (CD200R) expressed mostly on myeloid cells, which also has two extracellular IgSF domains but a longer cytoplasmic region with several tyrosine residues that can be phosphorylated [22]. Functional analysis suggests that the leukocyte CD200 protein can mediate a down-regulatory signal to myeloid cells through the inhibitory CD200R. Thus the CD200 null mice have an increased susceptibility to autoimmune disease induction and myeloid cells expressing CD200R are more activated [23]. CD200 and a viral homologue found in Kaposi sarcoma virus, when expressed at the cell surface, gave inhibition of production of inflammatory cytokines from activated macrophages [24]; and targeting the CD200-CD200R interaction with agonistic mAb or CD200-Fc fusion proteins *in vivo *ameliorates autoimmunity in disease models [25,26].

Protein arrays can be divided into two major classes: 'forward phase' if the analytes are captured from solution; or 'reverse phase' if the analytes are bound directly to the solid phase [27]. In forward phase protein microarrays, a bait molecule such as an antibody is immobilized onto a solid support to capture the analytes which can be proteins in purified form, or in complex solutions such as cell lysates [12] or tissue samples [27,28]. The bound analytes are detected either by direct labelling or via a secondary antibody. In reverse phase arrays, the analytes (typically purified proteins or cell lysates) are directly immobilized on the solid phase and antibodies or interacting proteins are applied in solution phase. The analytes can be labelled directly or detected using tags and signal amplification.

We have used the forward phase approach in the mapping of antigenic epitopes of hCD200R where different antibodies were immobilized on epoxy coated glass slides, incubated with the hCD200R analyte and detected with fluorescently labelled anti-CD200R antibodies. We have applied reverse phase arrays to three different purposes: -to test the reactivity of the fluorescently labelled mAb with directly immobilized hCD200R protein, -to map epitopes located near the ligand binding site using arrayed mutant hCD200R recombinant proteins and detection with fluorescently labelled mAb that block ligand-receptor interactions and -to detect the low affinity binding of immobilized CD200R to the multivalent CD200 ligand presented on fluorescently labelled beads. Our study extends the use of protein microarrays to the detection of transient cell surface protein interactions, which are of lower affinity than the reported cytokine arrays [29,30].

## Results and discussion

### Quantitative binding of DX147, DX136 and OX108 mAb to human CD200R

**Figure 1:**
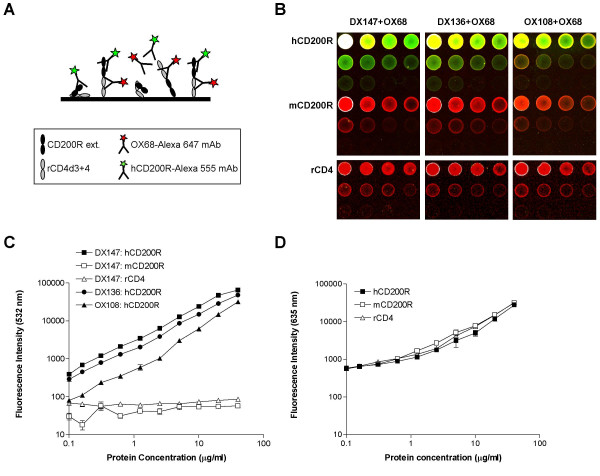
**Quantitative binding of mAb to hCD200R-CD4d3+4. **(A). Scheme illustrating a reverse phase microarray in which purified CD200R proteins were immobilized and then screened with fluorescent mAb specific for hCD200R or for the antigenic rCD4 tag (OX68) of the hybrid recombinant protein.(B). Typical microarray shows binding of OX68 mAb (red) to control proteins or the overlapping binding of hCD200R mAb and OX68 (yellow) to immobilized human CD200R.(C). Shows the green fluorescence intensity of each spot for all four replicates (mean ± SEM).(D). Shows red fluorescence intensity due to binding of OX68 mAb using data in left panel for DX147 (similar levels were found with the other mAb). Serial two-fold dilutions of purified, soluble, recombinant human and mouse CD200R-CD4d3+4 proteins, and of control rat CD4d3+4 were arrayed onto epoxy-coated microscope slides. Each protein dilution series was arrayed in 3 rows of 4 spots, ranging in concentration from 40 μg ml^-1 ^(first spot) to 0.08 μg ml^-1 ^(spot 10), with control spotting buffer containing 0.5 mg ml^-1 ^BSA in the last two spots. All arrays were performed in quadruplicate and a representative set is shown in (B). Each slide was incubated for 16 h at 4°C with a mixture of hCD200R mAb (DX147, DX136 or OX108) labelled with Alexa-555 (indicated as green fluorescence measured at 532 nm) and rCD4 mAb (OX68, detecting the antigenic tag and allowing for measurement of recombinant protein concentration) labelled with Alexa-647 (red fluorescence measured at 635 nm). At the highest concentrations, the hCD200R spots appear either white (saturating conditions) or yellow, due to the combination of green and red signals given by the specific binding of the Alexa-555-mAb to hCD200R and Alexa-647-OX68 mAb respectively. Quantitative measurements are expressed as mean fluorescence units at 532 nm (green) and 647 nm (red) versus amount of protein arrayed.

Purified, soluble recombinant hCD200R protein, engineered with domains 3 and 4 of rat CD4 as an antigenic tag (hCD200R-CD4d3+4) [31] was directly immobilized at different concentrations on epoxy-coated glass slides, in a reverse phase array as illustrated schematically (Fig. 1A). The hCD200R array was tested for reactivity with three different mAb: DX147, and two previously reported [31] mAb DX136 and OX108 able to block ligand binding. Controls on the arrays included recombinant mouse CD200R-CD4d3+4 protein (mCD200R) [31] and rat CD4d3+4. Figure 1B and 1C illustrate the strong and specific binding of all three fluorescently labelled mAb to hCD200R, as demonstrated by minimal reactivity with rCD4 (rat CD4d3+4) and lack of cross-reaction with mCD200R. DX147 gave the strongest labelling with linear binding from 0.08 to 20 μg ml^-1 ^mAb, reaching the upper limits of detection under the optimized voltage settings (65,000 units of green fluorescence) at 40 μg/ml (Fig. 1B). Binding of DX136 was linear over the full range of concentrations with a maximum binding of 48,000 units. OX108 bound more weakly, reaching a maximum value of 32,000 units. Sensitivity of detection, defined as two-fold binding above background, was estimated as the lowest hCD200R-CD4d3+4 concentration tested (0.08 μg ml^-1^) for DX136. A three-fold signal to noise ratio was achieved for DX147 at that concentration suggesting that sensitivity of detection was closer to 0.05 μg ml^-1^. For OX108, the limit of sensitivity was estimated at 0.3 μg ml^-1^. Thus DX147, DX136 and OX108 were able to detect 0.5 pg, 0.8 pg and 3.0 pg of hCD200R per spot respectively estimating a spot volume of 10 nl. The limit of sensitivity achieved was therefore between 8 and 50 attomol, assuming a molecular weight of 60,000 for hCD200R-CD4d3+4 protein. The amounts of human and mouse CD200R-CD4d3+4 and rCD4d3+4 protein on the microarray spots were similar as visualized by the red fluorescence of OX68 mAb recognising the CD4 tag present in each of the recombinant proteins (Fig. 1B and 1D). This indicates that the amount of protein detected is proportional to the amount arrayed in each spot and is highly reproducible. The limit of sensitivity of protein detection with Alexa 647-OX68 was approximately 0.3 μg ml^-1^, corresponding to 50 attomol of CD200R-CD4d3+4 proteins. Although not all molecules will be in a proper orientation for equal access to both anti-CD200R and anti-CD4, as illustrated in Fig. 1A, our results suggest that on average, there is a good correlation between the amount of specific antibody bound and the amount of protein arrayed.

### Orientation via antibody immobilization for epitope mapping on human CD200R

**Figure 2:**
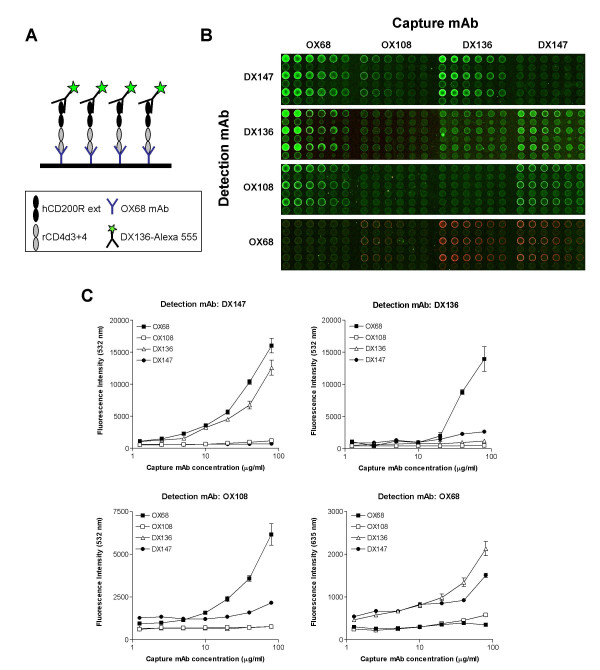
**Analysis of mAb reactivity with hCD200R by orientation via antibody immobilization. **(A). Scheme of one forward phase microarray in which purified human CD200R protein was immobilized via OX68 mAb and detected with the DX136 hCD200R mAb (green fluorescence).(B). Typical microarray shows binding of the hCD200R mAb (green) and OX68 mAb (red) to hCD200R immobilized via four different capture mAb.(C). Shows the mean fluorescence intensity ± SEM for each spot of all replicates. Serial two-fold dilutions of capture human CD200R mAb OX108, DX136 and DX147 and control rat CD4 mAb OX68 were arrayed onto epoxy-coated microscope slides. Each mAb dilution series was arrayed in quadruplicate of 2 rows of 6 spots, ranging in concentration from 80 μg ml^-1 ^(first spot) to 0.16 μg ml^-1 ^(spot 10), with control spotting buffer containing 0.5 mg ml^-1 ^BSA in the last two spots. The whole array was repeated on the slide for a total of 8 replicates per spot. Each slide was incubated for 2 h with 20 μg ml^-1 ^of purified recombinant hCD200R-CD4d3+4 protein, prior to incubation with Alexa-555-labeled CD200R mAb (DX147, DX136 or OX108) or Alexa-647 control rCD4 mAb (OX68). Quantitative measurements are expressed as mean fluorescence units at 532 nm (green) and 635 nm (red) versus amount of capture mAb arrayed.

We used forward phase protein microarrays to define the epitopes of hCD200R recognized by the three mAb introduced in the previous section. Serial dilutions of DX147, DX136 and OX108 mAb and the control CD4 mAb OX68 were directly immobilized on epoxy-coated glass slides as shown (Fig. 2A). The mAb arrayed act as capture reagents for hCD200R, used at a concentration of 20 μg ml^-1^. Each capture mAb binds to a different epitope on the hCD200R-rCD4d3+4 recombinant protein, thus orientating the protein on the array in a conformation that permits or restricts access to the same panel of mAb, used as detection reagents (Fig. 2A). Visual observation (Fig. 2B) and quantitative analysis (Fig. 2C) showed that DX147 reacts with an epitope spatially distinct from the more closely related DX136 and OX108 epitopes. As expected, OX68 is the most suitable capture mAb, as it binds the common CD4 tag allowing exposure of the two extracellular domains of hCD200R and binding of all three specific mAb. When the detection mAb is the same as the capture mAb, no significant binding above background is observed (Fig. 2C). DX147 binds specifically to hCD200R-CD4d3+4 orientated via OX68 and DX136 (16,021 and 12,570 green fluorescence units respectively at 80 μg ml^-1 ^of capture mAb), but not via OX108 or via itself. This is an indication that the binding epitopes for DX136 and DX147 are dissimilar. DX136 in turn, binds well to hCD200R-CD4d3+4 immobilized on OX68 (13,954 units of green fluorescence at 80 μg ml^-1^) and to a lesser degree to hCD200R-CD4d3+4 immobilized on DX147 (2,647 units), while not at all to hCD200R captured by OX108 (496 units). These data indicate that the DX136 epitope is spatially distinct from the DX147 epitope, but in close proximity to the OX108 epitope. This conclusion is substantiated by the lack of binding of OX108 mAb to hCD200R-CD4d3+4 captured on DX136. Orientating hCD200R via mAb OX68 allows for specific binding of all three anti-human CD200R mAb, in a linear fashion with limits of sensitivity of about 5 μg ml^-1 ^of immobilized mAb. The maximum amount of signal was obtained by capturing hCD200R with 80 μg ml^-1 ^of mAb OX68 and was equivalent to that observed by directly immobilizing approximately 10 μg ml^-1 ^hCD200R. OX68 is therefore the best mAb for capturing the chimaeric hCD200R-CD4 protein for optimal detection of hCD200R epitopes.

### Mapping of CD200R antigenic epitopes using mutants

Both CD200R and its ligand, CD200 contain two extracellular IgSF domains. The ligand-receptor interaction is therefore likely to occur in an end-to-end topology, requiring opposing cell surfaces to come into close proximity [22]. Previous studies have shown that the membrane distal N-terminal domain of CD200 is involved in binding its receptor [32]. In a recently published study [33], site directed mutagenesis was employed to map the ligand-binding domain of human CD200R using the structure of a typical Ig V domain, that of the human junctional adhesion molecule 1, JAM1 to predict the positions of out-pointing residues [34]. A panel of mutants was designed so as to target residues likely to be out-pointing from predictions of the beta strands of the N-terminal IgSF domain of hCD200R. The binding sites of CD200 and of the OX108 mAb known to block ligand interaction to the hCD200R mutants, were shown to be on the GFCC' face of the N-terminal IgSF domain [33].

**Figure 3:**
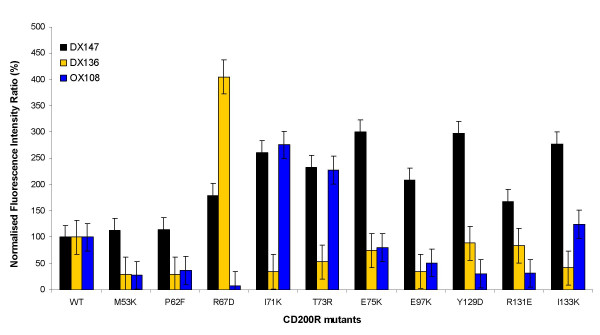
**Mapping of antigenic epitopes on hCD200R mutants. **The hCD200R mutants described in Table 1 and produced by transient transfection were arrayed (reverse phase) and tested for specific binding to mAb DX147, DX136 and OX108 and for reactivity with OX68 to quantify the relative amount of protein in each sample as described in Methods. Results for a panel of 10 mutants are plotted as percent antibody binding normalized to the wild type, non-mutated hCD200R protein (WT) values.

**Figure 4:**
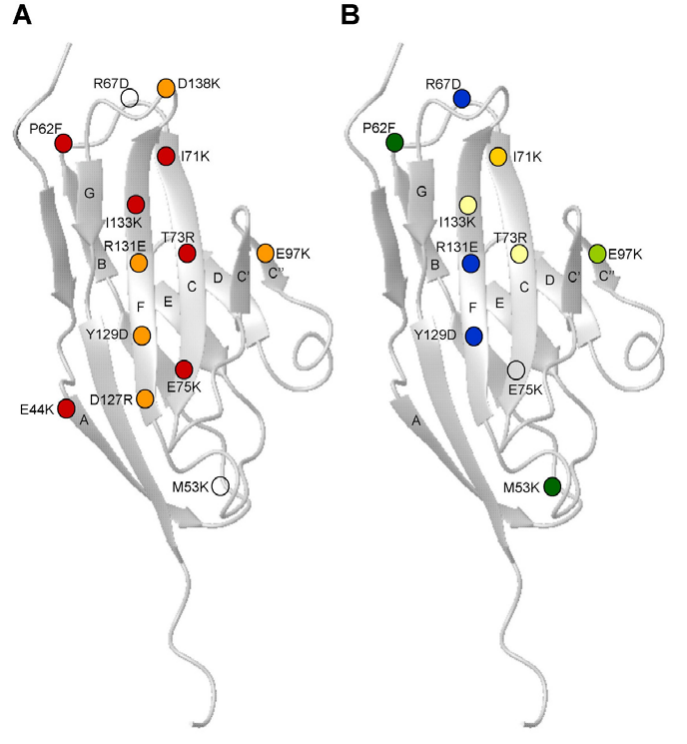
**Mapping of DX136 and OX108 epitopes on the N-terminal domain of human CD200R. **(A). The mutants giving complete or nearly complete inhibition of CD200 binding, determined by BIAcore analysis, are indicated with red circles (<35% binding compared to non-mutated WT hCD200R protein), whereas those giving partial effects (35–70% binding) are depicted in orange. Data from [33].(B). The mutants giving severe inhibition (<35% compared to WT) of OX108 and DX136 mAb binding, as determined by microarray analysis, are depicted in blue and yellow respectively. Dark green circles represent mutants that severely impaired both OX108 and DX136 mAb binding. Mutants partially affecting DX136 binding (35–50%) are shown in pale yellow. Mutants that severely affect DX136 binding (35% binding or less) but have only a partial effect on OX108 binding (50%) are represented in pale green. Open circles depict mutants that did not affect the binding of CD200, OX108 or DX136 mAb. The CD200R model is based on a typical Ig V domain from the human junctional adhesion molecule-1 (JAM1) [34]. The beta sheets are labelled with the GFC face orientated in front and the BED face behind.

The same panel of hCD200R-CD4d3+4 mutant proteins (Table 1) was analyzed by reverse protein microarrays (analogous to Fig. 1A) for binding to mAb DX147, DX136 and OX108 in order to map these epitopes. The mutant proteins were expressed by transient transfection in serum-free medium, concentrated and arrayed. Purified human and murine CD200R serving as positive and negative controls were immobilized at concentrations ranging from 0.08 to 40 μg ml^-1^. Lack of binding by a specific mutant or group of mutants is suggestive of the corresponding residues defining the location of the antigenic epitopes. None of the hCD200R mutants tested had lost binding to DX147, confirming unpublished data that this epitope lies, not in the N-terminal domain of CD200R but in the membrane proximal domain. Some of the mutants showed increased binding of mAb over the wild type despite these being normalized with OX68 (e.g. R67, I71K). This presumably reflects variations in epitope availability due to direct immobilization of the mutant proteins on the array, which are less likely to occur in BIAcore studies where mutants were immobilized via OX68 mAb [33]. Thus our data analysis was focused on mutants with major impairment in binding activity. Nine of the mutants showed reduced binding to either DX136 and/or OX108 mAb, relative to wild type hCD200R, as represented graphically in Figure 3. One mutant illustrated (E75K) did not affect binding in a significant manner. The position of these mutants is shown on the model of the N-terminal domain of hCD200R, based on a typical IgSF domain of similar size (Fig. 4). Panel A shows the mutants affecting CD200 binding, as reported in [33]. These mutants were mostly in the GFCC' face and in particular the F and C strands. Panel B shows the mutants with reduced binding to DX136 and/or OX108. It is immediately noticeable that mutants within the F and C strands also appear to have lost binding to DX136 and OX108 mAb, suggesting close proximity of these epitopes to the CD200 binding site, in agreement with the ligand blocking activity of these mAb. However, the key residues were not identical as shown by the differing effects of the mutants I71K and R67D. Furthermore, the microarray study confirms the results obtained by individual analysis of each mutant by surface plasmon resonance in terms of residues involved in OX108 binding; the most critical amino acid appears to be R67 located in the B-C loop [33]. The finding of mutants that had apparently gained CD200R mAb binding activity compared to the OX68 mAb recognising the CD4 tag, shows that spotting can have effects not seen by indirect methods such as the BIAcore. However there was very good correlation between available BIAcore data and microarray data. Thus this method provides a rapid high throughput method to identify the rare mutants that affect antigenic activity that can then be characterised further e.g. by BIAcore analysis.

**Table 1:** hCD200R protein mutants tested. Mutant proteins were constructed as described in [33], expressed by transient transfection in serum-free medium, concentrated and immobilized on epoxy-derivatized slides. The predicted positions of the residues are located in the modelled V-like N-terminal IgSF domain unless noted otherwise (C domain).

**#**	**Mutant Name**	**Predicted Position**
1	Wild-type	
2	D30K*	N-term
3	K40D*	A strand
4	L42E*	A strand
5	E44K*	A strand
6	E44A	A strand
7	E44D*	A strand
8	M53K	A-B loop
9	N56D	B strand
10	P62F	B strand
11	I64S*	B-C loop
12	R67D	B-C loop
13	I71K	C strand
14	T73R	C strand
15	E75K	C strand
16	R79E	C-C' loop
17	Q81K	C-C'
18	S83D*	C" strand
19	E97K	C" strand
20	T106D*	D strand
21	D116K*	D-E loop/ E strand
22	A123D	E strand
23	Y129D	F strand
24	R131E	F strand
25	I133K	F strand
26	D138K*	F-G loop
27	R143D*	F-G loop
28	H146D	G strand
29	Q148E*	G strand
30	L150D*	G strand
31	T156N*	A strand C domain
32	N160D	A-B loop C domain
33	A175D*	B-C loop C domain

* Mutants expressed with concentration below sensitivity threshold, for which no antibody binding data could be derived.

### Reactivity of human, rat and mouse CD200R with multimeric human CD200

**Figure 5:**
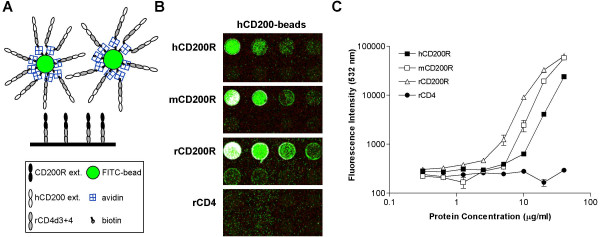
**Binding of multimeric CD200 ligands to CD200R proteins. **(A). Diagram of the reverse phase array depicting immobilized CD200R interacting with the multivalent bead ligand.(B). A representative microarray set showing binding of hCD200 beads to CD200R-CD4d3+4 proteins, but not control rat CD4.(C). Mean fluorescence intensity ± SEM of all four sets is shown versus receptor protein concentration arrayed. Serial two-fold dilutions of purified, soluble, recombinant human, mouse and rat CD200R-CD4d3+4 proteins, and of control rat CD4 were arrayed onto epoxy-coated microscope slides. Each receptor protein dilution series was arrayed in 3 rows of 4 spots, ranging in concentration from 40 μg ml^-1 ^(first spot) to 0.08 μg ml^-1 ^(spot 10), with control spotting buffer containing 0.5 mg ml^-1 ^BSA in the last two spots. Only the first 8 dilutions (2 rows) are shown in (B) and analyzed in (C). All arrays were performed in quadruplicate. All receptors were arrayed on the same slide, which was incubated for 16 h at 4°C with polyvalent human CD200-CD4d3+4 FITC-fluorescent beads. At the highest concentrations, the hCD200R spots appear white (saturating conditions). Quantitative measurements are expressed as fluorescence units at 532 nm (green) versus amount of protein arrayed.

The interaction of human CD200 with its receptor hCD200R is of low affinity, with a K_D _of ~0.5 μM at 37°C and *t*_1/2 _of 7 s [24], typical of the interaction of many leukocyte membrane proteins [15]. Such an interaction could not be detected when immobilized hCD200R was incubated with fluorescently labelled purified monomeric hCD200 protein (data not shown). In order to develop leukocyte membrane receptor-ligand microarray assays, high avidity detection reagents are required. Recombinant hCD200 protein was constructed by linking the extracellular domains of human CD200 with domains 3 and 4 of rat CD4 (CD4d3+4) as an antigenic tag. This construct contains a 19 amino acid sequence at the C-terminus of the protein, which can be enzymatically biotinylated on a specific lysine residue using the *E. coli *BirA enzyme [35]. Expression of the construct was demonstrated by inhibition of a rat CD4 ELISA using OX68 mAb. The recombinant protein bound the mAb OX104 (mouse anti-human CD200), indicating that it was antigenically active, as assessed by BIAcore analysis (data not shown) and its biotinylation was confirmed by streptavidin binding. The biotinylated hCD200-CD4d3+4 protein or the control CD4d3+4 protein were attached to avidin-coated FITC-fluorescent beads via their biotin tag, thus creating polyvalent CD200 and control reagents. These beads were used to detect specific binding of CD200 to human, mouse and rat CD200R proteins arrayed directly on epoxy-coated glass slides (Fig. 5A). Additional controls included rCD4d3+4 protein arrayed on the slide and tested for reactivity with both types of beads. Strong binding of hCD200-beads to all three CD200R proteins was observed (Fig. 5B and 5C) indicating that the proteins immobilized on the glass surface had retained their capacity to bind ligand with maximum mean values of 63,500 green fluorescence units for rat CD200R (saturating), 43,600 for mouse CD200R and 23,800 for human CD200R. The fluorescence appears granular as one is actually visualizing the small fluorescent beads. Binding was detected at concentrations of receptors ranging from approximately 5 to 40 μg ml^-1 ^corresponding to 1–8 femtomol per spot on the microarray. The non-specific binding of hCD200-beads to immobilized rCD4 protein was negligible (Fig. 5B and 5C) and control CD4d3+4 beads did not react with any of the arrayed proteins (data not shown). The multivalent hCD200-beads cross-reacted with rat and mouse CD200R as expected, as BIAcore analysis has shown that hCD200 interacts with human, rat and mouse CD200R with affinity constants within a log of each other [31]. Sensitivity of detection, defined as two-fold binding above background was achieved with concentrations of 5 μg ml^-1 ^for rat CD200R and 10 μg ml^-1 ^for human and murine CD200R. This corresponds to 1–2 femtomol of immobilized receptors interacting with the multimeric human CD200 ligand.

## Conclusion

In order to study interactions of leukocyte membrane proteins using high throughput microarray techniques, it was essential that the proteins be immobilized at low concentrations and in a biologically active form. It is critical that weak interactions between leukocyte membrane proteins be detected without interference by the anomalous binding due to denatured proteins, which is more of a contributing factor in the study of low affinity interactions. We first established that recombinant CD200R proteins could be immobilized directly in reverse phase arrays, in a conformation capable of reacting with three different mAb. We then demonstrated that recombinant mutant hCD200R proteins produced in transient expression systems were present in sufficient amounts to be immobilized directly and tested for reactivity with specific mAb, permitting mapping of epitopes. These data show that high throughput analysis of cell surface proteins can be achieved in reverse phase arrays using recombinant proteins derived from transient transfectants in a non-purified form. We also used forward phase arrays for competitive analysis of antibodies and mapping of their epitopes. This approach is valuable for rapidly screening antibody specificities and assessing protein orientation needed for optimal presentation of immunogenic determinants.

We also showed that binding of CD200 ligand to its cell surface receptor can be achieved by increasing the avidity of the reaction via coupling of the biotinylated recombinant CD200 protein to fluorescently labelled avidin coated beads. The fluorescent beads offer an efficient technology for the analysis of low affinity interactions typical of those observed for leukocyte membrane proteins and many other cellular proteins.

## Methods

### Materials

Monoclonal antibodies (mAb) DX147 (rat IgG1), and DX136 (rat IgG2a) to human CD200R were generously given by DNAX Research Institute (Palo Alto, CA). The mAb OX108 (mouse IgG1) to human CD200R [31] and OX68 (mouse IgG1) to rat CD4 domains 3 and 4 (rCD4d3+4) have been described previously [36].

### Recombinant proteins

The soluble biotinylated forms of human, mouse and rat CD200R were produced as described [22,31,36]. Briefly, the entire extracellular region of human, mouse or rat CD200R was amplified by PCR and cloned in the pEF-BOS-CD4d3+4bio-XB vector [35]. These constructs were then subcloned into the expression vector pEE14, and stably secreting CHO.K1 cell lines were established [37]. Human, mouse or rat CD200R-CD4d3+4 proteins were purified from the tissue culture medium by immunoaffinity chromatography with OX68 mAb-Sepharose 4B that recognizes the CD4 protein tag [36]. Prior to use, the purified CD200R proteins were fractionated by gel filtration on Superdex S-200 (Pharmacia, Uppsala, Sweden) to exclude protein aggregates. The soluble, biotinylated form of human CD200 was produced in an identical fashion, by subcloning the amplified extracellular region of human CD200 [38] using *Xba*I/*Sal*I digestion to the pEF-BOS-CD4d3+4bio-XB vector [36]. This construct was used to transfect HEK293T cells using the calcium phosphate method. The protein expressed was enzymatically biotinylated and used to generate multivalent binding reagents by coupling to avidin-coated fluorescein isothiocyanate (FITC)-loaded beads (Spherotech Inc., Libertyville, IL) as described previously [35].

Mutants of human CD200R (hCD200R) were prepared as described [33]. Briefly, the mutations were introduced by site directed mutagenesis using PCR and two mutagenic oligonucleotides into a construct comprising the extracellular domains of human CD200R together with domains 3 and 4 from rat CD4 (CD4d3+4) as an antigenic tag. The mutants were transiently expressed in HEK 293T cells using X-VIVO 10 media (BioWhittaker, Nottingham), concentrated about 10 fold and levels of expressed protein quantified by ELISA. This media contains 1 mg ml^-1 ^BSA so after concentration the final protein concentration is around 10 mg/ml.

### Antibody labelling

Purified antibodies were dialysed against PBS prior to labelling with Alexa Fluor 555 or Alexa Fluor 647 fluorescent amine-reactive dyes using the Molecular Probes Monoclonal Antibody Labelling Kits (Cat. No. A-20186 and A-20187) and according to the manufacturer's instructions (Molecular Probes, Invitrogen Ltd.). Labelling reactions were carried out using 100 μg of IgG and yielded labelled proteins ranging in concentration from 1 to 4 × 10^-6 ^M. The degree of conjugation was estimated at 2–4 moles of dye per mole protein. Labelled antibodies were stable for up to 2 months at 4°C.

### Preparation of microarrays

Protein solutions to be arrayed were prepared in 96 well plates and 12 μl aliquots were transferred to single wells of Genetix 7020, 384-well plates (Genetix Ltd, New Milton, UK). Concentrations tested ranged from 0–80 μg/ml and all dilutions were performed in Protein Array Spotting Solution (Genetix) with the addition of 0.5 mg/ml BSA and 0.02% NaN_3_. A QArrayMini microarray printer (Genetix) was used to apply the protein solutions onto epoxy-coated microscope slides using 300 μm solid tipped tungsten microarraying pins (Genetix). Preliminary experiments established the printing conditions with fluorescently labeled OX68 mAb. Of several types of slides tested, the epoxy-coated ones were the best in terms of spot morphology, cost and reproducibility and were used in all subsequent experiments.

Most array designs were performed using 8 pins to obtain spots with a 440 μm diameter and centre-to-centre spot spacing of 700 μm in both directions. Source plates were kept at 8°C, and a 65% average internal humidity was maintained. After printing, the slides were left in the arraying chamber for 30–60 minutes under the same conditions. The slides were then washed using the Protein Array Processing Kit (Genetix Ltd; stored at 4°C and the solutions supplemented with 0.02 % NaN3) by inversion for 1 min in Clean Up Buffer (Genetix) to remove unbound proteins and incubation for 30 min in Blocking Buffer. Slides were washed 3 times in PBS, once in H_2_O to remove excess salt and dried using an air brush, and stored at 4°C, with desiccant in a sealed slide box. Preliminary experiments were done by forward phase arrays to establish optimal conditions. OX68 mAb (100 μg ml^-1^ to 20 μg ml^-1^) was immobilized, incubated with rat CD4 at 5 μg ml^-1^ 0.1 mg ml^-1^ BSA, washed and incubated with labeled W3/25 mAb (5 μg/ml) and linearity of detection demonstrated. Specificity was also shown by the fact that OX68 was not reactive with CD4 immobilised on OX68 and vice versa.

### Labelling of microarrays

Slides were placed in hybridization chambers (Corning Incorporated, UK) and the humidification wells filled. LifterSlips, (Erie Scientific, Portsmouth, USA) were placed gently over the marked boundaries of the arrays and the binding reagent (25–70 μl) was introduced with a micropipette. In experiments where CD200R-CD4 hybrid proteins (including the mutant studies) were arrayed, Alexa-555 anti-CD200R antibodies (mAb DX147, DX136 or OX108; 5–10 μg ml^-1^) were added to measure the amount of specific antibody bound and Alexa-647-CD4 mAb (OX68 5–10 μg ml^-1^) was included to assess the amount of hybrid protein present. In experiments where capture antibodies were arrayed (Fig. 2), a 2 h incubation with purified protein, such as CD200R-CD4d3+4 or CD4d3+4 at 20 μg ml^-1^, was performed prior to incubation with the detection antibodies. In the experiments detecting CD200 (ligand) binding to immobilized CD200R (Fig. 5), arrays were incubated with biotinylated hCD200-CD4d3+4 streptavidin-FITC beads. Incubations with detection reagents were carried out for 16 h at 4°C unless otherwise stated. The slides were immersed upside down in PBS/0.05% Tween-20, washed thrice with copious amounts of PBS/Tween-20, alternating shaking up and down under liquid in the Copeland jar and gentle rocking for 5–10 min each, followed by PBS (twice for 5 min) and a final H_2_O rinse. All washes were at room temperature and repeated following each incubation. After drying the slides with an air brush, the arrays were scanned using a GenePix4000B microarray scanner (Axon Laboratories, Palo Alto, CA) scanner using 532 nm and 635 nm lasers using the GenePix Pro 5.0 (Axon Laboratories) software. The PMT values were 720 and 1000 (532 nm and 635 nm respectively) for Figure 1, 780 and 950 for Figure 2, 900 and 1000 for Figure 3 and 850 for Figure 5 (532 nm only).

### Data analysis

All samples were tested in quadruplicate and all experiments repeated several times. The amount of antibody or ligand bound to the arrayed proteins and the amount of protein present in each spot were determined by comparing the fluorescence intensities read at 532 and 635 nm. Extraction of spot intensity data was performed using GenePix Pro 5.0 (Axon Laboratories) and ScanArray Express (Perkin Elmer). The background, calculated as the median of pixel intensities from the local area around each spot, was subtracted from the mean pixel intensity within each spot. To graphically represent the data, the values of the background-subtracted signal intensities were plotted against the known concentration of the protein spotted in the array. Sensitivity of detection for each spot was defined as a signal to noise ratio (S/N) of two-fold above background. S/N was calculated as: S/N = (background-subtracted median signal intensity) / (standard deviation of background signal intensity).

In the case of the mutant hCD200R proteins generated from culture supernatants of transient transfections, where protein concentration is unknown, the background-subtracted values for both 532 and 635 nm-signal intensities were corrected for internal protein signal by subtracting the corresponding value of a "mock transfectant" spot. The corrected values for the red channel (representing the amount of protein assessed from the CD4 content) were normalised to 100% with respect to the wild-type hCD200R transient transfection sample. All hCD200R mutants with red channel values below 50% were assumed to contain insufficient amount of protein and were excluded from the analysis. The green channel background-subtracted, "mock"- transfectant corrected values (G) were divided by the corresponding red channel ones (R) to correct for variations in the amount of expressed protein contained in each individual spot (G/R ratio). Finally, the G/R ratio was normalized to 100% with respect to the hCD200R-CD4d3+4 wild-type protein, before graphical representation.

## List of abbreviations

IgSF, immunoglobulin superfamily; hCD200, human CD200; CD200-CD4d3+4, chimaeric recombinant CD200 protein with rat CD4 domains 3+4; hCD200R, human CD200 receptor; mCD200R, mouse CD200 receptor; rCD200R, rat CD200 receptor; SPR, surface plasmon resonance; WT, wild type.

## Authors' contributions

ML set up and developed the protein arrays with DV specialising in the experiments with the multimeric beads and data analysis. Both wrote the paper and DV prepared the figures. DH produced the mutant CD200R proteins and advised on their analysis. MF produced the mAb OX108 and the human, mouse and rat CD200R proteins. NS provided expertise in setting up the system for protein microarrays and their basic analysis. NB devised the initial project, advised throughout and helped with the manuscript writing.
